# The clinical and radiological characteristics of pulmonary cryptococcosis in immunocompetent and immunocompromised patients

**DOI:** 10.1186/s12890-021-01630-3

**Published:** 2021-08-13

**Authors:** Yan Hu, Si-Ying Ren, Peng Xiao, Feng-Lei Yu, Wen-Liang Liu

**Affiliations:** 1 Department of Thoracic Surgery, The Second Xiangya Hospital of Central South University, No. 139 Renmin Road, Changsha, 410011 China; 2 Department of Respiratory and Critical Care Medicine, The Second Xiangya Hospital of Central South University, Changsha, 410011 China; 3 Department of Cardiothoracic Surgery, The Third Xiangya Hospital of Central South University, Changsha, 410013 China

**Keywords:** Pulmonary cryptococcosis, Computed tomography, Fungal infection, Immune status, Cavitation

## Abstract

**Background:**

We characterized the clinical features, radiographic characteristics, and response to treatment of immunocompetent and immunocompromised patients with pulmonary cryptococcosis (PC).

**Methods:**

We retrospectively reviewed the medical records and radiological profiles of patients diagnosed with PC who received surgical resection between May 2015 and November 2020 in a tertiary referral center.

**Results:**

A total of 21 males and 18 females were included in the study. 23 patients were immunocompetent and 20 out of the 39 were asymptomatic. Immunocompetent patients were diagnosed with PC at a younger age than immunocompromised patients (48.9 vs 57.1 years, *P* = 0.02). Single nodule pattern was the most frequent lesion pattern (33 out of 39, 84.6%) and right upper lobe was the most common site of location (15 out of 47, 31.9%). The majority of lesions were located peripherally (38 out of 47, 80.9%) and most lesions were 1–2 cm in diameter (30 out of 47, 63.8%). Cavitation was more likely to occur in immunocompromised patients (5 out of 11, 45.5%) than in immunocompetent patients (6 out of 36, 16.7%) (*P* = 0.04) and there was complete resolution of PC in all patients treated with anti-fungal therapy.

**Conclusions:**

Immunocompetent patients were diagnosed with PC at a younger age than immunocompromised patients. Single nodule pattern was the most frequent lesion pattern in PC patients. Cavitation was more likely to occur in immunocompromised patients than in immunocompetent patients.

## Background

Pulmonary Cryptococcosis (PC) is a pulmonary fungal disease caused by the inhalation of Cryptoccocus neoformans or Cryptococcus gattii spores into the respiratory system [1]. It is well-known that PC mostly occurs in immunocompromised patients, including AIDS patients, patients with hematologic malignancies, organ transplantation recipients, and patients receiving immunosuppressive agents [2, 3]. However, the incidence of PC in immunocompetent patients has increased in recent years [4, 5]. Radiographically, PC may appear as a solitary nodule or mass, multifocal nodules, pulmonary consolidations, or bronchopneumonia [6, 7]. It is difficult to differentiate PC from primary lung cancer, tuberculosis, or metastasis especially when PC presents as a solitary pulmonary nodule. The aim of this retrospective study is to characterize the clinical features, radiographic characteristics, and response to treatment of immunocompetent and immunocompromised patients with PC.

## Methods

### Study subjects

We retrospectively reviewed the medical records and radiological profiles of patients diagnosed with PC who received surgical resection in our center between May 2015 and November 2020. Patients with PC would be included in the study if they met all of the following conditions: 1. presence of pulmonary nodule or mass in which lung cancer could not be ruled out clinically; 2. PC was histologically and microbiologically confirmed; 3. clinical data and CT imaging information were available.

The following clinical data were extracted from the medical records: sex, age, presenting symptoms, cormorbidities, smoking status, surgical and antifungal therapy, pathology, follow-up, and prognostic outcomes. Chest CT images were analyzed and the location, pattern, size, margins, pleural sign, and presence of air bronchogram, cavitation, Halo sign, and mediastinal lymphoadenopathy were recorded. Positron emission tomography (PET)/CT images were evaluated for standardized uptake values (SUVs) if accessible. Pulmonary Lesions or lymph nodes with higher FDG uptake was defined when their maximum SUV (SUV_max_) was higher than that in the normal lung tissues or the mediastinal blood pool, respectively. Diagnosis of PC was confirmed by postoperative pathology. All samples were formalin-fixed, paraffin-embedded, and stained with haematoxylin–eosin (H&E), Gomori methenamine silver (GMS), and periodic acid-Schiff (PAS). The Institutional Review Boards of Second Xiangya Hospital approved the study.

### Statistical analysis

Descriptive analyses of patient demographics, clinical features, radiological findings, pathological data, and treatment outcomes were performed. All data were presented as mean ± SD for continuous variables, and as numbers with percentages for categorical variables. The Student’s test was used for continuous variables and Pearson χ^2^ test or Fisher’s exact test for categorical variables. All statistical analyses were performed using STATA software. *P* < 0.05 was considered to indicate statistical significance.

## Results

### Patient demographics and clinical features

A total of 21 males (53.8%) and 18 females (46.2%) were included in the study. The mean age was 51.1 years (standard deviation: 10.9). There were 32 non-smokers and seven current or ex-smokers. Of the 39 patients, 27 were asymptomatic (69.2%), and presented with an incidental finding of pulmonary lesions following routine health examinations. In the other 12 patients, symptoms including cough (n = 6), sputum (n = 6), chest pain (n = 4), chest distress (n = 3), hemoptysis (n = 1), and fever (n = 1) were observed. In terms of comorbidities, nine patients had hypertension, eight had a malignancy, two had previous environmental exposures (a clear history of exposure to sulfides (n = 1) or coal (n = 1)), one had severe diabetes mellitus, one received bilateral kidney transplantation, and one had a previous history of pulmonary tuberculosis. For host immune status, 29 patients were immunocompetent. The other 10 patients were considered immunocompromised, including eight patients with a malignancy, one with organ transplantation and diabetes mellitus, and one with tuberculosis receiving antituberculosis drugs. Immunocompetent patients were diagnosed with PC at a younger age than immunocompromised patients (48.9 vs 57.1 years, *P* = 0.02). None of the patients had AIDS at the time of diagnosis of PC. 38 patients were diagnosed with PC using surgical modality and only one patient was diagnosed using percutaneous translung biopsy before surgery. Type of surgery included wedge resection (n = 22), segmentectomy (n = 3), and lobectomy (n = 14). The initial suspected diagnosis before surgical resection of the lesion(s) was primary lung cancer in 20 lesions, pulmonary metastasis in five lesions, non-specific granulomatous disease in six lesions, pulmonary tuberculosis in two lesions, infectious disease in two lesions, benign lesions in nine lesions, and hamartoma in two lesions. Immunocompromised patients were more likely to obtain initial diagnosis of pulmonary metastasis than immunocompetent patients (*P* = 0.001). Patient demographic and clinical information is summarized in Table 1.

**Table 1:** Demographics and clinical features of patients with PC

Clinical characteristics	Total (n = 39)	Immunocompetent patients(n = 29)	Immunocompromised patients(n = 10)	P values
Gender (male)	21	16	5	0.78
Current or ex-smoker	7	4	3	0.25
Clinical symptoms				
Cough	6	4	2	0.64
Sputum	6	4	2	0.64
Chest pain	4	2	2	0.24
Chest distress	3	2	1	0.75
Hemoptysis	1	1	0	0.55
Fever	1	0	1	0.08
Comorbidities				
Malignancy	8	0	8	
Environmental exposures	2	2	0	
Diabetes mellitus	2	0	2	
History of tuberculosis	1	0	1	
History of organ transplantation	1	0	1	
Diagnosis by				
Surgery	38	29	9	
Percutaneous translung biopsy	1	0	1	
Type of surgery				0.19
Wedge resection	22	18	4	
Lobectomy	14	10	4	
Segmentectomy	3	1	2	
Initial diagnosis before surgery				
Primary lung cancer	20	18	2	0.06
Pulmonary metastasis	5	0	5	0.001
Non-specific granulomatous disease	6	5	1	0.68
Pulmonary tuberculosis	2	2	0	0.42
Infectious disease	2	2	0	0.42
Benign lesions	9	7	2	0.93
Pulmonary cryptococcosis	1	0	1	0.06
Hamartoma	2	2	0	0.42

### Radiologic presentation findings

**Fig. 1:**
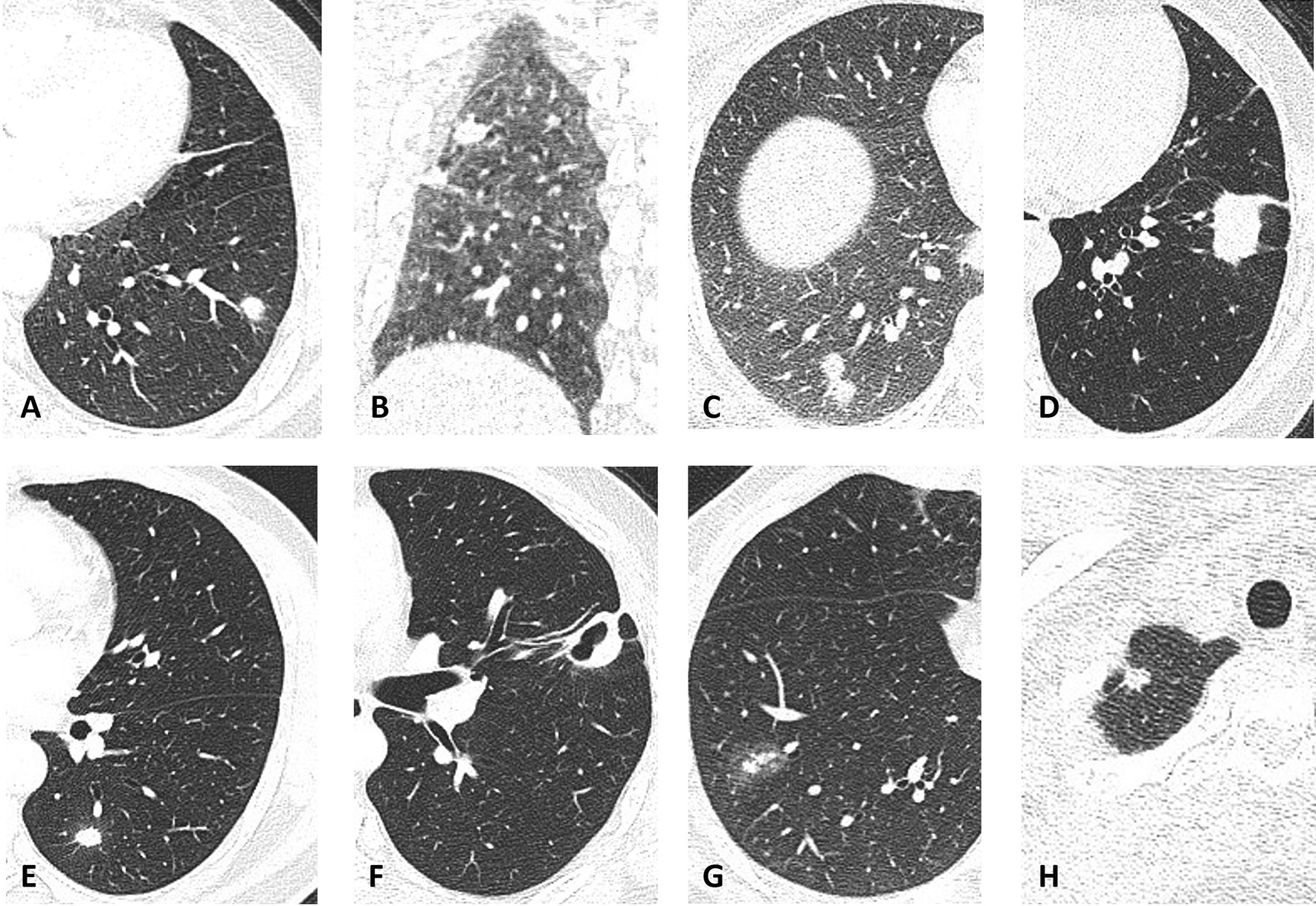
Representative radiological findings of pulmonary cryptococcosis. **a** Single nodular pattern of the lesion in the left lower lobe of a 51-year-old immunocompetent woman; **b** Scattered nodular pattern of the lesion in the right lower lobe of a 52-year-old immunocompetent man; **c** Clustered nodular pattern of the lesion in the right lower lobe of a 40-year-old immunocompetent woman; **d** Mass-like pattern of the lesion in the left lower lobe of a 66-year-old immunocompromised woman; **e** Spiculation of the lesion in the left lower lobe of a 51-year-old immunocompetent woman; **f** Cavitation of the lesion in the left upper lobe of a 41-year-old immunocompetent man; **g** Halo sign of the lesion in the right lower lobe of a 49-year-old immunocompetent man; **h** Pleural indentation of the lesion in the right upper lobe of a 44-year-old immunocompetent woman

The thoracic CT findings are summarized in Table 2. Single nodule pattern was the most frequent lesion pattern, presenting in 33 of the 39 (84.6%) patients, followed by scattered nodule (7.7%), clustered nodule (5.1%), and mass-like (2.5%) (Fig. 1). Pleural effusion was not seen in any patient and mediastinal lymph node enlargement was observed in 10 patients. Most lesions were located in the right upper lobe (15 lesions, 31.9%), followed by right lower lobe (13 lesions, 27.6%) and left lower lobe (13 lesions, 27.6%). Pulmonary lesions (38 out of 47, 80.9%) were located mostly in the peripheral lung field (outer third of the lung). The diameter of the lung lesions was less than 1 cm in nine lesions (19.1%), 1–2 cm in 30 lesions (63.8%), and greater than 2 cm in eight lesions (17%). 20 (42.6%) lesions were spiculated. Air bronchogram was noted in three lesions (6.4%), halo sign in nine (19.1%), and pleural indentation in 10 (21.3%). Cavitation was noted in 11 lesions and was more likely to occur in immunocompromised patients (5 out of 11, 45.5%) than in immunocompetent patients (6 out of 36, 16.7%) (*P* = 0.04).

**Table 2:** Thoracic CT findings of patients with PC

CT Findings	Total patient(n = 39)	Immunocompetent patients(n = 29)	Immunocompromised patients(n = 10)	*P* value
Lesion pattern				0.29
Single nodule	33	25	8	
Scattered nodule	3	2	1	
Clustered nodule	2	2	0	
Mass-like	1	0	1	
Pleural effusion	0	0	0	
Mediastinal lymphoadenopathy	10	6	4	0.23
Location of the lesions	Total lesions(n = 47)			0.06
RUL	15	12	3	
RML	1	0	1	
RLL	13	12	1	
LUL	5	2	3	
LLL	13	10	3	
Distribution of the lesions				0.43
Peripheral	38	30	8	
Central location	9	6	3	
Diameter of the lesions				0.71
< 1 cm	9	6	3	
1–2 cm	30	24	6	
> 2 cm	8	6	2	
Spiculation	20	16	4	0.64
Air bronchogram	3	3	0	0.32
Cavitation	11	6	5	0.04
Halo sign	9	7	2	0.93
Pleural indentation	10	8	2	0.77

The PET/CT results are summarized in Table 3. A total of 14 patients underwent PET/CT scans. SUV_max_ ranged from 1.8 to 43.5 (average 9.12 ± 10.46, median 6.3). All of the patients showed higher FDG uptake in the pulmonary lesions and four patients revealed higher uptake in lymph node areas. There was no significant difference in SUV_max_ between immunocompetent and immunocompromised patients (*P* = 0.49).

**Table 3:** PET/CT results of patients with PC

PETCT findings	Total (n = 14)	Immunocompetent patients (n = 7)	Immunocompromised patients (n = 7)	*P* value
SUVmax	9.12 ± 10.46	11.16 ± 14.58	7.1 ± 3.85	0.49
Pulmonary lesions with higher uptake	14	7	7	
Lymph nodes with higher uptake	4	1	3	

### Pathological diagnosis

**Fig. 2:**
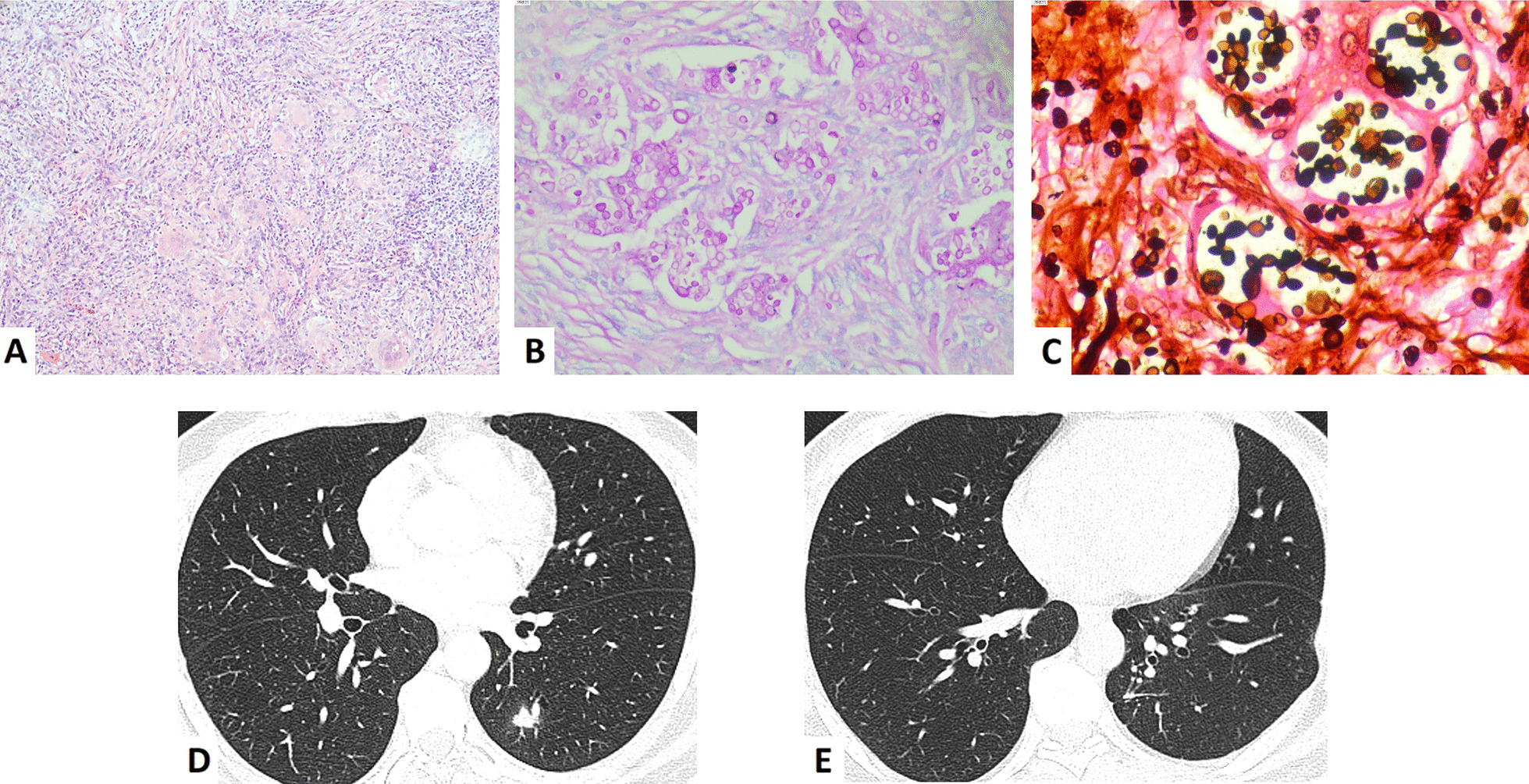
Histology and perioperative CT findings of an immunocompetent patient with pulmonary cryptococcosis. **a** granulomas were formed and round cryptococcal spores were seen using H&E staining (× 100); **b** Narrow-based budding yeasts surrounded by thick capsules in the lung tissue were positive using PAS staining (× 200); **c** Narrow-based budding yeasts surrounded by thick capsules in the lung tissue were positive using GMS staining (× 400); **d** Preoperative CT image showing a solitary nodule in the left lower lobe; **e** Postoperative CT image at 21 months follow-up showing no local relapse after wedge resection of the nodule. H&E, haematoxylin–eosin; GMS, Gomori methenamine silver; PAS, periodic acid-Schiff

Diagnosis of PC was confirmed in all patients by postoperative pathology. H&E staining showed a variety of inflammatory reactions from inflammation to well-formed granulomas, accompanied by various degrees of fibrosis and necrosis (Fig. 2). The presence of the cryptococcal pathogen was identified in all samples by histochemically staining with Gomori methenamine silver (GMS) and periodic acid-Schiff (PAS).

### Treatment and prognosis

The follow-up period for the patients ranged from 2.5 to 48 months (mean, 11.8 months). Of the 38 patients who were diagnosed by surgery, one patient was lost to follow up and the remaining 37 patients received postoperative antifungal therapy (fluconazole 400 mg/day). The duration of the antifungal therapy ranged from 2 weeks to 8 months (mean, 3.5 months). One patient obtained the diagnosis of PC before surgery. She received oral fluconazole (400 mg/day) for 6 months preoperatively and 3 months postoperatively. All of the 38 patients who received follow-up were completely cured after treatment.

## Discussion

PC is an important opportunistic fungal disease in immunocompromised patients, but it is also increasingly identified in immunocompetent patients [1]. Previous studies have shown that immunocompetent patients were diagnosed with PC at a younger age than immunocompromised patient [2, 3, 8]. This finding was also seen in our study, which showed that immunocompetent patients were eight years younger than immunocompromised patients (48.9 vs 57.1 years, P = 0.02). PC generally presents with a variety of clinical presentations, from asymptomatic to nonspecific symptoms including cough, sputum, and chest pain [9–11]. In the present study, 27 patients were asymptomatic and the other 12 patients presented with a variety of symptoms such as cough (n = 6), sputum (n = 6), chest pain (n = 4), chest distress (n = 3), hemoptysis (n = 1), and fever (n = 1). Therefore, the variability in the clinical presentation of PC means that it is not possible to confidently confirm the diagnosis based on clinical characteristics alone [12].

The characteristics of chest CT findings in PC has been well described in the literature [13, 14]. The radiological presentations of PC varied from a solitary pulmonary nodule or mass, multiple nodules, lobular, segmental or subsegmental consolidation, to bronchopneumonia [1, 6]. Several studies reported that single nodule pattern [6, 9, 13, 15] and peripheral distribution of lesions [3, 6, 8, 11, 16] were the most common pulmonary CT findings. These observations were supported by our study, which showed that the single nodule pattern was the most commonly identified radiological pattern (33 out of 39, 84.6%) and that most lesions (38 out of 47, 80.9%) were located in the peripheral lung field. PC may tend to involve the lower lobes [4, 9], specifically the right lower lobe [6, 8, 11]. However, a study by Sui et al. reported that PC was not associated with any particular lung lobe [13]. In the present study, we found that PC was most commonly located in the right upper lobe, followed by the right lower lobe and left lower lobe.

It is believed that cavitation suggests a long-term focal lung abnormality [2]. Patients with cavitary lesions probably undergo a more severe cryptococcal infection that requires a more aggressive antifungal therapy [2]. Several studies observed that immunocompromised patients were more likely to develop severe pulmonary abnormalities characterized by cavitation than immunocompetent patients [2, 9, 13]. Our study showed similar findings. Higher FDG uptake often occurs in PC lesions [6, 11, 13, 15, 17]. A study by Wang et al. reported that PC lung lesions and mediastinal lymph nodes with increased uptake were seen in 37 and 8 out of 42 PC patients, respectively [6]. In our study, all of the 14 patients who underwent PET/CT showed higher FDG uptake in the pulmonary lesions, and four patients had higher FDG uptake in lymph node areas.

The treatment approach to cryptococcal infection depends on the host immune status, the extent of the disease, the presence of symptoms, and whether the infection is localized to the lungs or central nerve system, or whether it is disseminated [18–20]. Most PC infections in immunocompetent patients are treated with fluconazole, with the addition of amphotericin B in immunocompromised patients, CNS Cryptococcus, or those with diffuse pulmonary disease [21]. However, the management of PC in immunocompetent patients is uncertain [5]. Several previous studies have reported the effectiveness of antifungal therapy alone in the successful treatment of PC [5, 8, 22]. Studies by Nadrous et al. [22] and Zhang et al. [11] reported that simple observation without antifungal therapy may be enough for treating asymptomatic immunocompetent patients with isolated PC, as the lung lesions seen in these patients may undergo spontaneous remission.

Surgical resection is a key component of the modern diagnosis and treatment of fungal lung diseases [5]. Surgery not only affords reliable diagnosis but also represents a treatment modality for many patients with PC, and it may be the optimal management approach for those patients who have undiagnosed isolated PC lesions. Previous studies have shown no relapse of PC after surgery [6, 9, 13, 15]. In addition, whether adjuvant antifungal therapy may be required after surgery remains to be determined. Yang et al. suggested no need for additional antifungals by showing that 15 patients underwent surgical resection alone and no patient who underwent surgical resection developed progressive disease [5]. However, this evidence should be considered cautiously because of the limited sample size and short follow-up durations in their study. In the present study, aside from one patient who was lost to follow-up, all of the other (n = 38) patients received postoperative antifungal therapy, and all of these patients showed no evidence of relapse with a mean follow-up of 11.8 months.

There were several limitations to our study. Firstly, this was a single-center retrospective study by design, and therefore may be associated with selection bias. Secondly, immunocompetent and immunocompromised patients were grouped according to their known pre-existing comorbidities/medical history. Immune function testing would be required to definitively establish the immune status of included patients [11]. Thirdly, the number of PC patients included in the study was relatively small. Future large-scale study with longer-term follow-up is warranted.

In conclusion, immunocompetent patients were diagnosed with PC at a younger age than immunocompromised patients. Single nodule pattern was the most frequent lesion pattern in PC patients. Cavitation was more likely to occur in immunocompromised patients than in immunocompetent patients.

## Data Availability

The data that support the findings of this study are available from the corresponding author upon reasonable request.

## Abbreviations

PC: Pulmonary cryptococcosis
PET: Positron emission tomography
SUVs: Standardized uptake values
